# A randomized controlled trial to evaluate the impact of a geo-specific poster compared to a general poster for effecting change in perceived threat and intention to avoid drowning ‘hotspots’ among children of migrant workers: evidence from Ningbo, China

**DOI:** 10.1186/s12889-017-4462-x

**Published:** 2017-05-30

**Authors:** Yinchao Zhu, Xiaoqi Feng, Hui Li, Yaqin Huang, Jieping Chen, Guozhang Xu

**Affiliations:** 1 Institute of Non-Communicable Diseases Control and Prevention, Ningbo Municipal Center for Disease Control and Prevention, No. 237, Yongfeng Road, Haishu District, Ningbo City, 315010 People’s Republic of China; 2 Population Wellbeing and Environment Research Lab (PowerLab), School of Health and Society, Faculty of Social Sciences, University of Wollongong, Northfields Avenue, Wollongong, Australia; 3 Early Start Research Institute, Faculty of Social Sciences, University of Wollongong, Northfields Avenue, Wollongong, Australia; 4 Department of Health Surveillance, Jiangbei District Center for Disease Control and Prevention, No. 466, Qingjiang Road, Jiangbei District, Ningbo City, 315020 People’s Republic of China

**Keywords:** Effect evaluation, Drowning, Migrant children, China

## Abstract

**Background:**

Drowning among children of migrant workers is a major, though neglected public health issue in China.

**Methods:**

A randomised controlled trial was used to examine the potential impact of viewing a preventive health poster with/without geo-located drowning events on perceptions of drowning risk among Chinese migrant children. A total of 752 children from three schools in Jiangbei district were selected by multi-stage sampling and randomly assigned to the intervention (*n* = 380) or control (*n* = 372). Multilevel models were used to analyse changes in responses to the following questions after viewing the assigned poster for 10 min: (1) *“Do you believe that drowning is a serious health problem in Ningbo city?”*; (2) *“Do you believe that there are lots of drowning-risk waters around you?”;* (3) *“Do you believe that the likelihood of your accessing a drowning-risk water is great?”*; and (4) *“Would you intend to avoid accessing to those drowning-risk waters when being exposed?”*

**Results:**

At baseline there were no significant differences between the intervention and control groups in perceptions of drowning risk or covariates. Following the intervention, participants that viewed the geo-specific poster were more likely to respond more favourably to the first three questions (*p* < 0.001) than those who viewed the standard poster. However, there was no substantive difference between the geo-specific or standard poster in terms of changing intentions to avoid drowning hotspots (*p* = 0.214).

**Conclusions:**

Use of ‘geo-located’ information added value to the effectiveness of a drowning prevention poster for enhancing awareness of drowning hotspots among children of migrant workers.

**Trial registration:**

Chinese Clinical Trial Registry ChiCTR-IOR-16008979 (Retrospectively registered) (The date of trial registration: Aug 5, 2016, the date of enrolment of the first participant: Nov 10, 2015).

## Background

Evidence from the Global Burden of Disease study of 2013 shows that drowning, a preventable cause of death, remains common despite a notable decline since 1990 [1]. It has been highlighted by the World Health Organization as a neglected public health concern [2, 3].Part of the problem may stem from differential risk, with the majority of deaths occurring among children [3, 4]. Child mortality attributable to drowning in China is in the range of 6–8 deaths per 100,000 people, making drowning the first leading cause of death among the domestic children aged 1–14 years [5]. In China, an additional layer of complexity is the higher risk of drowning among children of migrant workers compared to their locally-born peers [6, 7]. Rapid economic development has resulted in unprecedented levels of migration of people to China’s eastern cities and dramatic urbanisation; increasingly as families [8, 9]. Children of migrant workers, however, not only must contend with an unfamiliar context often without the constant support of their parents, who are often blamed for children’s injuries [10], but they must also negotiate a rapidly changing built environment that brings new risks that migrant parents are perhaps not always equipped to manage on their own.

**Fig. 1:**
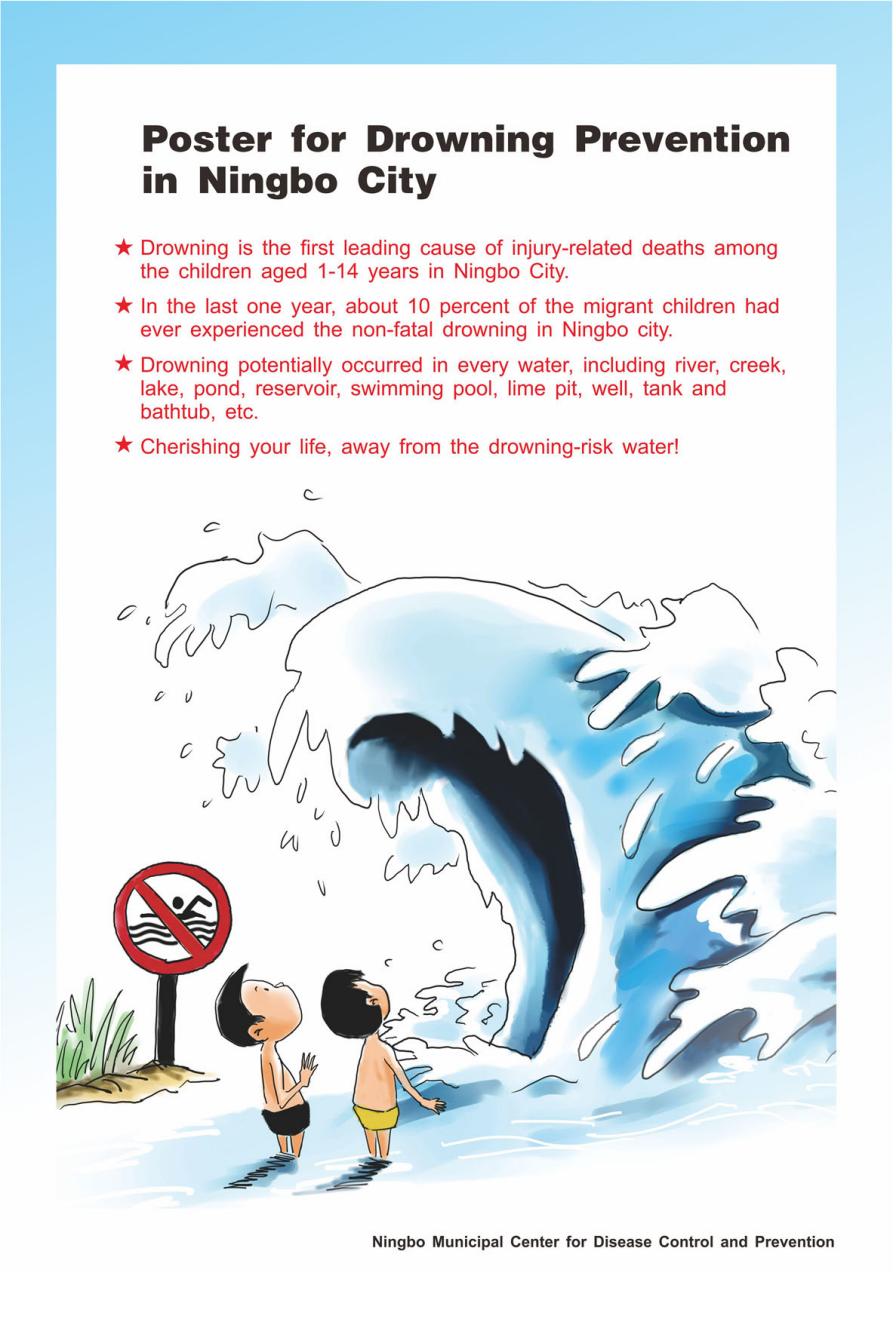
The previous drowning prevention poster without geo-located drowning events

**Fig. 2:**
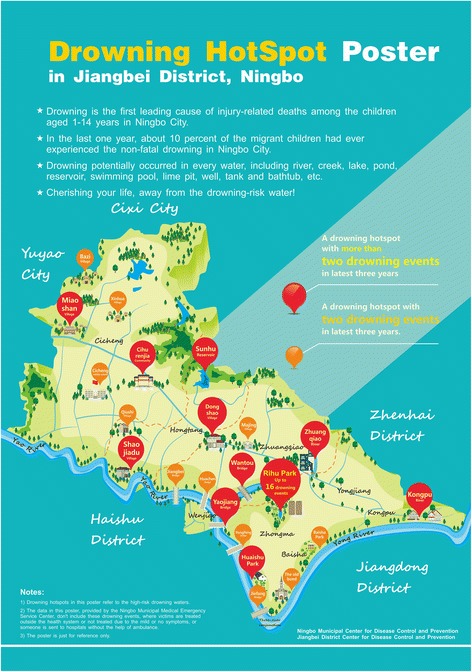
The new drowning prevention poster with geo-located drowning events inserted here

Recognising the children of China’s migrant workers as a high risk group for mortality by drowning, the Ningbo Municipal Center for Disease Control and Prevention (CDC) initiated a three-year programme to enhance drowning prevention in Jiangbei district in 2013. Previous drowning prevention programmes had utilised prevention posters as a means for instilling general warning messages on what children should do or not do (e.g. Fig. 1). The new programme reasoned that in a new built environment, a better understanding of where aquatic hazards are geographically could add potential value for drowning prevention among children of migrant workers. Accordingly, a new poster that mapped drowning ‘hotspots’ was developed [11], utilizing data from the Ningbo Medical Emergency Service System (see Fig. 2). In this study, we conducted an experiment to test whether the new ‘geo-located’ poster enhanced perceptions of drowning threat and strengthened intentions to avoid drowning ‘hotspots’, compared to a previous drowning prevention poster, among a group of children from migrant worker families living in Jiangbei district, Ningbo city.

## Methods

### Design

The experiment was conducted using a cluster randomised controlled trial in November 2015. The impact of viewing a preventive health poster with/without geo-located drowning events on perceptions of drowning risk among Chinese migrant children was evaluated. Participants who met the inclusion criteria in this study were randomized on the level of class to one of two groups by using a random number generated in Microsoft Excel. The experiment was scheduled with all participants under the coordination of Jiangbei District Education Bureau. The study design and protocol was approved by the ethics committee of Ningbo CDC.

### Participants and sample

The study population was restricted to children in schools, who had resided in Jiangbei district for at least 6 months, which is a central urban district in Ningbo, which in turn is a mid-coastal city of China containing 0.24 million local population and 0.19 million migrant population [12].Schools were selected based upon several criteria. Firstly, these schools were specifically for the floating children in Jiangbei District. Secondly, there were various types of schools, including elementary school only (for grade of 1–6 only), junior middle school only (for grade of 7–9 only), elementary and secondary school (for grade of 1–9 fully). We selected one school for each type by using a random number generator.

Participants were in grades4 to 9.Participants were excluded if they did not consent to participate in this study or absented on the survey day for sick/compassionate leave.

Owing to the originality of two tested posters in this study and a lack of other relevant evidence, the sample size was determined based on the lowest variability in prevalence of intentions to avoid drowning ‘hotspots’ (9.02% and 5.10% respectively for two groups) from the pilot study. Written informed consent was obtained from a guardian of each participant prior to the investigation. Three schools in Jiangbei district were selected randomly from a list of 9 schools specifically for children of migrant workers. Two classes were randomly selected from each grade of 4 to 9 inclusive (age ranged 9 to 17). One class from each grade was randomly assigned to the intervention, with the other allocated the control.

### Interventions

The ‘geo-located’ poster and a general drowning prevention poster were especially displayed for the intervention and control group respectively. Poster viewing lasted approximately 10 min, after which the posters were withdrawn by trained staff from Ningbo CDC serving as research assistants on the study. The viewing of each poster was made consistent by ensuring that the bottom of each poster was fixed as high as 1.2 m on the wall, in front of which enough space was left for students to observe freely. The maximum number of participants for each evaluation was 15. Free discussion among students was allowed during observation, but their teachers were not permitted any involvement.

### Measurement and definitions

The purpose and instructions for the experiment were all explained to each class prior to commencing the baseline survey. The children in the intervention and control classes were surveyed on their perceptions of drowning threat and intentions to avoid drowning ‘hotspots’ pre- and post-viewing of the assigned poster. The questions in each survey were identical, except for information on demographics that was only collected at baseline. The four belief questions were designed with guidance from the Health Belief Model [13]. Perceived threat of drowning was assessed using the question: (1) *“Do you believe that drowning is a serious health problem in Ningbo city?”*; and (2) *“Do you believe that there are lots of drowning-risk waters around you?”.* Attitude towards visiting drowning ‘hotspots’ was assessed using the questions: (3) *“Do you believe that the likelihood of your accessing a drowning-risk water is great?”*; and (4) *“Would you intend to avoid accessing to those drowning-risk waters when being exposed?”* For each question, responses were recoded dichotomously with “definitely /probably not” = “no” and “definitely /probably yes” = “yes”.

### Data analysis

The data were entered into Epidata 3.0 software (The EpiData Association”, Odense, Denmark) and statistical analyses were conducted using Stata v.14 (Stata Corp, College Station, TX) and MLwiN [14]. Cross-tabulations and Chi-square tests were used to describe the balance of the intervention and control groups according to gender and school grade in the study sample. The percent of each group responding positively (i.e. yes) to the outcome variables were reported at baseline and follow-up for the intervention and control groups separately. Positive transition referred to the participants’ belief conversion from “no” at baseline to “yes” at follow-up, and negative transition referred to a change in belief in the opposite direction. McNemar tests were used to examine change in outcomes within the intervention and control groups. Multilevel logistic regressions fitted with a two-way interaction between time and experimental grouping were used to assess the impact of the intervention on each outcome variable. For the first three outcomes, ‘yes’ was modelled as the response category. For the fourth outcome (on drowning hotspot avoidance), ‘no’ was modelled as the response category since the majority of the sample said ‘yes’. Multilevel models were fitted with person at level 2 and observation at level 1, as initial three-level models fitted with school at level 3 were not found to add value, as the between-school variances were not significant. This was in line with expectations since there were only three schools and this ‘n’ would normally prohibit accurate variance estimation. Nonetheless, two-level multilevel models were still applied to account for correlated responses within individuals between baseline and follow-up. Parameters in the multilevel logistic regressions were expressed as odds ratios with 95% confidence intervals. All tests were two-sided and a *p*-value of <0.05 was set as the level of statistical significance.

## Results

A total of 752 students from 24 classes were invited to participate in the study. Owning to the immediate review of reclaimed questionnaires by the research assistants, all 752 responding questionnaires were valid without any missing information. Of the 752 participants, 380 (50.53%) entered into the intervention group and 372 (49.47%) in the control group. Demographic characteristics are reported in Table 1. There was good comparability in demographic characteristics between each group and also no significant difference in beliefs relating to drowning prevention at baseline (Table 1).

**Table 1:** Description of the study sample

		Control	Intervention	Chi-Square	*p*-value
N		372	380		
Do you believe that drowning is a serious health problem in Ningbo city?	No	70.70	73.42		
	Yes	29.30	26.58	0.692	0.405
Do you believe that there are lots of drowning-risk waters around you?	No	73.66	78.42		
	Yes	26.34	21.58	2.344	0.126
Do you believe that the likelihood of your accessing a drowning-risk water is great?	No	83.87	87.89		
	Yes	16.13	12.11	2.514	0.113
Would you intend to avoid accessing to those drowning-risk waters when being exposed?	No	21.24	18.42		
	Yes	78.76	81.58	0.938	0.333
Gender	Boys	46.51	45.00		
	Girls	53.49	55.00	0.172	0.679
Age Group	9-11y	43.02	42.89		
	12-13y	28.49	29.74		
	14-17y	28.49	27.37	0.036	0.982
School Grade	4	18.82	19.47		
	5	19.35	18.95		
	6	18.01	17.89		
	7	13.44	14.21		
	8	15.59	14.47		
	9	14.78	15.00	0.303	0.998
School	1	32.26	32.63		
	2	22.85	23.16		
	3	44.89	44.21	0.036	0.982

Positive responses (see Table 2) before and after poster viewing for the intervention group was 55.79%(up from 26.58%, McNemar test χ^2^ = 97.016, *p* < 0.001) for belief in drowning is a serious health problem in Ningbo, 50.00% (up from 21.58%, χ^2^ = 87.045, *p* < 0.001) for belief in the existing of drowning-risk waters nearby, 29.21% (up from 12.11%, χ^2^ = 43.557, *p* < 0.001) for belief that coming into contact with a drowning-risk water is great, and 89.47% (up from 81.58%, χ^2^ = 28.125,*p* = 0.044) for intention to avoid accessing drowning-risk waters. Positive responses for the control group to the same questions respectively were 36.29% (up from 29.30%, χ^2^ = 26.000, *p* < 0.001), 31.18% (up from 26.34%, χ^2^ = 13.500, *p* < 0.001), 20.16% (up from 16.13%, χ^2^ = 9.000, *p* = 0.003) and 83.33% (up from 78.76%, χ^2^ = 217.000,*p* < 0.001).The percentages of belief positive transition for the four questions in the intervention group were significantly higher than their peers in the control group(Chi-square test,χ^2^ = 71.474, *p* < 0.001; χ^2^ = 84.222, *p* < 0.001; χ^2^ = 41.076, *p* < 0.001;χ^2^ = 4.050, *p* = 0.044)(Table 2).

**Table 2:** Outcome variable change over time between the intervention and control groups for the study sample

Outcome variable	Intervention (*n* = 380)				Control (*n* = 372)				Chi-Square	*p*-value
	N: (%)				N: (%)					
	Positive transition from baseline to follow-up	Responded positively at baseline and follow-up	Negative transition from baseline to follow-up	Responded negatively at baseline and follow-up	Positive transition from baseline to follow-up	Responded positively at baseline and follow-up	Negative transition from baseline to follow-up	Responded negatively at baseline and follow-up		
Do you believe that drowning is a serious health problem in Ningbo city?	31.32	24.47	2.11	42.11	6.99	29.30	0.00	63.71	71.474	<0.001
Do you believe that there are lots of drowning-risk waters around you?	31.84	18.16	3.42	46.58	5.65	25.54	0.81	68.01	84.222	<0.001
Do you believe that the likelihood of your accessing a drowning-risk water is great?	21.32	7.89	4.21	66.58	5.38	14.78	1.34	78.49	41.076	<0.001
Would you intend to avoid accessing to those drowning-risk waters when being exposed?	8.16	81.32	0.26	10.26	4.57	78.76	0.00	16.67	4.050	0.044

**Fig. 3:**
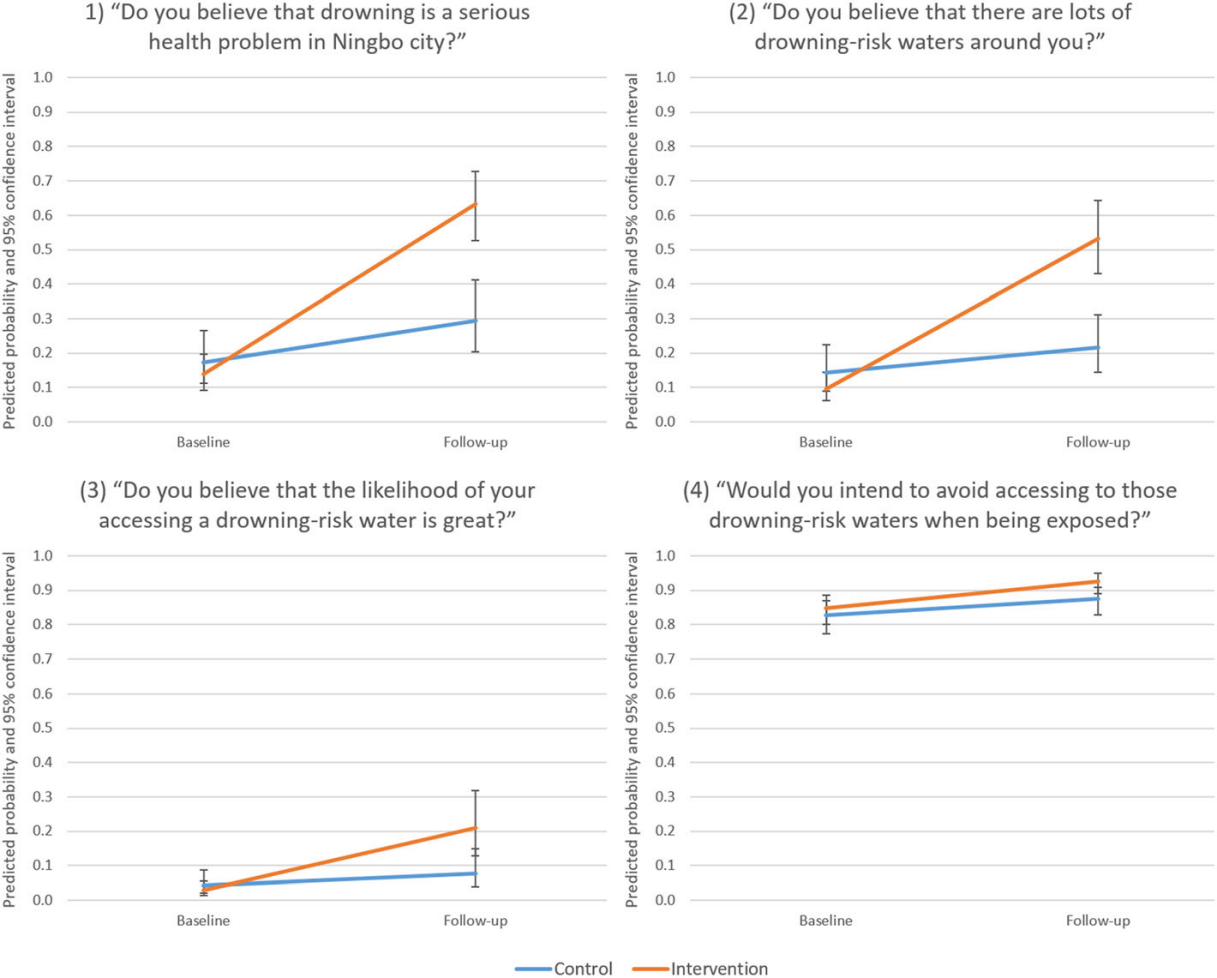
The predicted probabilities within the multilevel logistic regressions for the four outcome variables inserted here

In multilevel logistic regressions, variance partition coefficients of 61.3%, 60.3%, 72.3% and 45.6% indicated substantial amounts of the overall variation in each outcome manifested between participants (with the remainder manifesting as variation over time within participants). As balance in covariates was achieved through randomisation, no further adjustment was required. A two-way interaction between time and intervention was found to be significantly associated with the odds of three of the four outcome variables (*p* < 0.001) (Table 3). At baseline, no significant differences between the intervention and control groups were observed. Among the control group, the odds of reporting a positive compared to negative response over time increased significantly for the questions concerning the belief that drowning is a serious health problem in Ningbo (OR = 1.99, *p* = 0.005) and that there are lots of drowning-risk waters nearby (OR = 1.64, *p* = 0.045). No significant differences in odds were observed over time among the control group with respect to the questions of access and avoidance of drowning-risk waters. In contrast, the interaction indicated the ‘geo-located’ poster had a differential impact in strengthening awareness of drowning as a serious health problem in Ningbo (OR = 5.41, *p* < 0.001), on potential threat of drowning ‘hotspots’ (OR = 6.60, *p* < 0.001) and potential access to drowning ‘hotspots’ (OR = 4.89, *p* < 0.001), but not on intentions to avoid drowning hotspots (OR = 1.42, *p* = 0.214). The predicted probabilities from the interactions within each of the multilevel logistic regressions are illustrated in Fig. 3.

**Table 3:** Assessment of the intervention effect using multilevel logistic regression

	Do you believe that drowning is a serious health problem in Ningbo city? (Yes)	Do you believe that there are lots of drowning-risk waters around you? (Yes)	Do you believe that the likelihood of your accessing a drowning-risk water is great? (Yes)	Would you intend to avoid accessing to those drowning-risk waters when being exposed? (Yes)
Odds Ratio (95% Confidence Interval)				
Experiment (ref: Control)				
Intervention	0.76 (0.38, 1.51)	0.63 (0.31, 1.28)	0.65 (0.24, 1.74)	1.19 (0.78, 1.84)
*p*-value	0.435	0.204	0.391	0.420
Time (ref: Baseline)				
Follow-up	1.99 (1.24, 3.19)	1.64 (1.01, 2.67)	1.86 (0.99, 3.49)	1.35 (0.93, 1.95)
*p*-value	0.005	0.047	0.054	0.112
Time × Experiment				
Baseline × Intervention	5.41 (2.76, 10.59)	6.60 (3.33, 13.10)	4.89 (2.05, 11.68)	1.42 (0.82, 2.49)
*p*-value	<0.001	<0.001	<0.001	0.214

## Discussion

Evidence-based measures to enhance the prevention of drowning among children per se, but especially those of migrant workers in Chinese cities, are a public health imperative. The key finding of this study is that the use of a map to visualise the locations of drowning ‘hotspots’ is more effective than a standard drowning prevention poster at enhancing awareness and perceived threat of drowning ‘hotspots’. The enhanced poster did not, however, appear to significantly increase intentions to avoid drowning ‘hotspots’. To our knowledge, this is the first study to examine this issue in the case of drowning prevention among children of China’s migrant workforce.

Although previous work on drowning prevention is thin, the findings are consistent with the impact of tobacco-related health warning images on urging smokers’ thoughts of quitting smoking [15, 16]. There is one point of departure, however, in terms of delivering the message. Imagery depicting the unfavourable health outcomes resulting from tobacco smoke is known to have shock value [17]. The intervention tested in this study did not contain equivalent imagery. Instead, the geographic visualisation of drowning ‘hotspots’ made the difference. This approach is reasoned to be more compatible with Chinese culture of indirection and euphemism [18].

The importance of this result in the context of children of migrant workers in China ought to be underlined, but also caveated. It is known that the children of migrant workers are at a greater risk of drowning [19]. These children often experience radical changes in living context, such as geography, weather, social culture, built environment and living without constant supervision from parents may directly contribute to drowning risk [19–22]. Measures are needed which recognise that parents are not solely to blame for the occurrence of child drowning, but in fact that this is a systemic public health issue requiring multi-sectoral action. Increasing child knowledge of drowning ‘hotspots’ can enhance their awareness of local hazards.

Although awareness of drowning ‘hotspots’ was increased, it is interesting to note that the intervention did not appear to alter participants intentions to avoid those areas. An important factor to take into account is the very high levels of intent to avoid drowning-risk areas at baseline for the intervention and control groups. This means there may be a ‘hard core’ minority of participants who stated that they were not intending to avoid drowning-risk waters and that the viewing of a poster did not change that intent. It is unclear at this stage what factors underpinned this result, but it seems unlikely that intentions to avoid drowning ‘hotspots’ remain unchanged despite an increase in knowledge of the issue. Rather, it is already well known that awareness and education on health issues is a necessary but insufficient condition for driving behavioural change. It may be, for instance, that characteristics of local built and physical environments where the children live and attend school make it very challenging or virtually impossible for them to contemplate avoiding drowning ‘hotspots’ (perhaps because of the proximity of the hotspots relative to where their family home is). As with other forms of behavioural change (e.g. smoking cessation) it is clear that education and awareness is a necessary but insufficient condition. Further investigation is warranted to better understand why stated intentions to avoid drowning ‘hotspots’ did not appear to shift as much as awareness and perceptions of threat, in order to determine the extent that the drivers of those intentions are themselves modifiable.

There are two main caveats. First, the study showed differences in responses between intervention and control participants within a short space of time. Follow-up research is needed to examine whether the impact is sustained over a longer period. Second, the outcomes in this study are stated preferences. They inform but are no substitutes for revealed preference data. Should this intervention be scaled up, it is important for future work to use appropriate mortality surveillance data and relevant methodologies (e.g. an interrupted time series model) to examine whether switching to a ‘geo-located’ format decreases the number of drownings which occur among children of migrant workers in China.

## Conclusions

This study provided evidence to suggest that involving geographic visualisation in a drowning prevention poster adds value towards enhancing child awareness and perceptions of threat in relation to drowning ‘hotspots’. The poster did not make a statistically significant difference to stated intentions to avoid drowning ‘hotspots’. Aside from most participants already stating that they intended to avoid drowning-risk waters, for the minority who did not this may be partially because a poster is insufficient to shift their behaviour, but it may also be because of constraints relating to mobility in relation to drowning hazards situated within local built and physical environment (e.g. near where they live).Long-term follow-up and evaluation of a scaled up initiative on drowning occurrence are essential next steps for enhancing the prevention of this neglected public health issue.

## Abbreviations

CDC: Center for Disease Control and Prevention

## References

[1] Haagsma JA, Graetz N, Bolliger I, Naghavi M, Higashi H, Mullany EC, et al. The global burden of injury: incidence, mortality, disability-adjusted life years and time trends from the global burden of Disease study 2013. Inj Prev. 2016;22(1):3–18. 10.1136/injuryprev-2015-041616 PMC4752630 26635210

[2] World Health Organization (2014). Global report on drowning: preventing a leading killer.

[3] World Health Organization. Drowning. 2016. http://www.who.int/mediacentre/factsheets/fs347/en/. Accessed Feb 2016.

[4] Centers for Disease Control and Prevention (2012). Drowning--United States, 2005-2009. MMWR Morb Mortal Wkly rep.

[5] Wang SY (2011). Epidemiological features and research progress of injury in China. Chin J Epidemiol..

[6] Zhu XX, Chen K, Liu QM, Shi WY, Xiang HQ, Fang SY, et al. Study on the risk factors of injuries among children at school age, from the families of migrant workers in Hangzhou city. Chin J Epidemiol. 2009;30(9):911–4. 20193226

[7] Zhu Y, Xu G, Li H, Huang Y, Ding K, Chen J (2015). Epidemiology and risk factors for nonfatal drowning in the migrant children. Southeast Asian J Trop med Public Health.

[8] National Population and Family Planning Commission (2012). Report on China’s migrant population development 2012.

[9] Gong P, Liang S, Carlton EJ, Jiang Q, Wu J, Wang L, et al. Urbanisation and health in China. Lancet. 2012;379(9818):843–52. 10.1016/S0140-6736(11)61878-3 PMC3733467 22386037

[10] Schnitzer PG, Dowd MD, Kruse RL, Morrongiello BA (2014). Supervision and risk of unintentional injury in young children. Inj Prev.

[11] Shenoi RP, Levine N, Jones JL, Frost MH, Koerner CE, Fraser JJ (2015). Spatial analysis of paediatric swimming pool submersions by housing type. Inj Prev..

[12] Ningbo Municipal Bureau of Statistics, China Bureau of Statistics Ningbo Investigation Team (2015). 2015 Ningbo Statisticall YearBook.

[13] Salazar LF, Crosby RA, Noar SM, Walker JH, Diclemente RJ, Diclemente RJ, Salazar LF, Crosby RA (2013). Models based on perceived threat and fear Appleas. Health behavior theory for public health principles, foundations, and applications.

[14] Rasbash J, Browne WJ, Goldstein H (2004). A User’s guide to MLwiN.

[15] Chang FC, Chung CH, Yu PT, Chao KY (2011). The impact of graphic cigarette warning labels and smoke-free law on health awareness and thoughts of quitting in Taiwan. Health Educ res.

[16] Fathelrahman AI, Omar M, Awang R, Borland R, Fong GT, Hammond D, et al. Smokers’ responses toward cigarette pack warning labels in predicting quit intention, stage of change, and self-efficacy. Nicotine Tob Res. 2009;11(3):248–53. 10.1093/ntr/ntn029 PMC2666375 19246625

[17] Wu D, Yang T, Cottrell RR, Zhou H, Yang XY, Zhang Y. The effects of tobacco-related health-warning images on intention to quit smoking among urban Chinese smokers. Health Educ J. http://journals.sagepub.com/doi/abs/10.1177/0017896914535377.

[18] Tingzhong Y (2010). Tobacco control theory and Implemetation.

[19] Schyllander J, Janson S, Nyberg C, Eriksson U, Stark B, Ekman D (2013). Case analyses of all children's drowning deaths occurring in Sweden 1998-2007. Scand J Public Health.

[20] Wang H, Smith GA, Stallones L, Xiang H (2010). Injury-related childhood mortality in migrant households in a southern city of China. Inj Prev.

[21] Franklin RC, Scarr JP (2014). Drowning- prevention, rescue, treatment.

[22] Sevilla-Godínez RE, Gómez-Lomelí ZM, Chávez-Ponce B, Orozco-Valerio M, Celis-de la Rosa A (2010). Prevalence of risk factors for drowning at home related to the socioeconomic level. Rev Med Inst Mex Seguro Soc.

